# Facile synthesis of pyrazolopyridine pharmaceuticals under mild conditions using an algin-functionalized silica-based magnetic nanocatalyst (Alg@SBA-15/Fe_3_O_4_)†

**DOI:** 10.1039/d2ra07228a

**Published:** 2023-04-03

**Authors:** Fereshte Hassanzadeh-Afruzi, Zeinab Amiri-Khamakani, Mahdi Saeidirad, Mohammad Mehdi Salehi, Reza Taheri-Ledari, Ali Maleki

**Affiliations:** a Catalysts and Organic Synthesis Research Laboratory, Department of Chemistry, Iran University of Science and Technology Tehran 16846-13114 Iran maleki@iust.ac.ir +98-21-73021584 +98-21-73228313

## Abstract

Pyrazolopyridines are common scaffolds in various bioactive compounds, which have several therapeutic effects and unique pharmacological properties. In this study, we fabricated a novel environmentally friendly silica-based nanocomposite as a multifunctional catalytic system for the synthesis of pyrazolopyridine derivatives. This novel heterogeneous nanocomposite named Alg@SBA-15/Fe_3_O_4_ (Alg stands for alginic acid), was prepared in several steps. In this regard, SBA-15 was synthesized by the hydrothermal method. Next, it was magnetized by Fe_3_O_4_ nanoparticles *via* an *in situ* co-precipitation process. Then, SBA-15/Fe_3_O_4_ particles were functionalized with 3-minopropyltriethoxysilane (APTES). Afterward, Alg@SBA-15/Fe_3_O_4_ was obtained by a nucleophilic substitution reaction between SBA-15/Fe_3_O_4_–NH_2_ and an as-synthesized methyl-esterified alginic. Different analyses such as Fourier-transform infrared (FTIR), energy-dispersive X-ray (EDX) spectroscopy, field-emission scanning-electron microscopy (FESEM), vibrating-sample magnetometer (VSM), X-ray diffraction (XRD), thermogravimetric analysis (TGA), and BET (Brunauer–Emmett–Teller) have been used to confirm the structure of the fabricated catalyst. The magnetic properties of the Alg@SBA-15/Fe_3_O_4_ catalytic system imparted by Fe_3_O_4_ MNPs enable it to be conveniently isolated from the reaction mixture by using an external magnet. According to the obtained results, the prepared nanocatalyst has high thermal stability and it lost approximately 26% of its weight up to 800 °C. Interestingly, a small amount of prepared nanocatalyst (0.02 g) has shown excellent catalytic performance in the synthesis of pyrazolopyridine derivatives (90–97%) in a short reaction time (20–30 min) at room temperature which can be attributed to its porous structure and large surface area, and the presence of many acidic and basic functional groups. In general, it can be argued that the Alg@SBA-15/Fe_3_O_4_ nanocomposite deserves more attention due to its non-toxicity, ease of preparation, good recyclability, and its high catalytic efficiency.

## Abstract 2

Pyrazolopyridines are common scaffolds in various bioactive compounds, which have several therapeutic effects and unique pharmacological properties.

## Graphical abstract

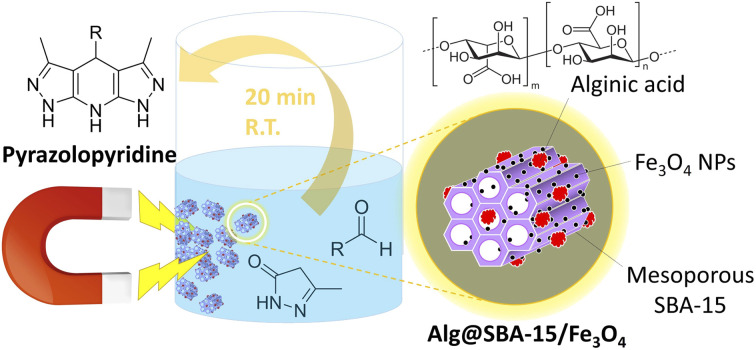

## 1. Introduction

It has been stated that nitrogen-containing heterocycles account for more than 90% of substances examined by pharmaceutical companies.^1^ Pyrazopyridine as a kind of nitrogen-rich heterocycle has attracted a lot of interest due to having valuable pharmacological and biological activities, including anti-virus^2^ and anti-leishmania,^3^ as well as anti-malarial^4^ properties. Tracazolate, cartazolate, and etazolate are some of pyrazolopyridine derivatives that are used as anxiolytic drugs. Other pyrazolopyridine-containing biologically-active compounds involve a GSK-3 inhibitor, and BAY 41-2272 can be utilized as cardiovascular therapeutic agents.^5^ Although various multi-step procedures for the construction of pyrazolopyridines have been developed in recent years, some of them suffer from one or more challenges for instance long reaction time, low yields, laborious work-up procedure, as well as generation of by-products. It seems that the development of efficient methods for the synthesis of pyrazolopyridines is still an important topic in chemistry because of their extensive biological activities. Multicomponent reactions (MCRs) is a practical approach to addressing the challenges of the pyrazolopyridine synthesis. MCRs are one-pot processes in which three or more reactants are reacted to form a particular product containing all of the reactants' atoms.^6,7^ These reactions have been broadly utilized for the synthesis of complicated bio-active organic compounds, particularly heterocyclic materials for instance the pyrazolopyridine, owing to their outstanding advantages such as atom economy, experimental simplicity, and the formation of several bonds in a single operation.^8^ The use of magnetic nanocatalysts in MCRs is an attractive idea for the development of sustainable methods in green synthetic chemistry.^9–11^

Porous materials with outstanding properties such as, uniform pore size, adjustable porous structure, and high surface area have piqued the curiosity of scientists.^12^ According to the IUPAC classification, porous materials can be categorized as microporous materials, whose pore width is less than 2 nm, mesoporous materials, whose pore width is 2–50 nm, and macroporous materials, whose pore width is more than 50 nm.^13^ Mesoporous materials were first discovered by Mobil Research and Development Corporation in 1992, and their synthesis and application have received great attention since then.^14^ Zhao and coworkers at the University of California, Santa Barbara reported the SBA-15 mesoporous silica for the first time in 1998.^13,15–17^ Well-defined pore size between 4 to 30 nm, thick pore walls, pore architecture,^11,18^ wide surface area (up to 1000 m^2^ g^−1^), high thermal and hydrothermal stability, and excellent capability to be modified/functionalized are among the features that make materials based on SBA-15 have different applications, including adsorption, drug delivery, imaging, sensor, and catalysis.^13,19–22^ One-pot synthesis or co-condensation, and grafting technique or post-synthesis are the two main methods used to modify/functionalize silica mesoporous materials.^13^ Incorporation of Fe_3_O_4_ magnetic nanoparticles as a super-paramagnetic substance into silica-based mesoporous supports is a very practical step towards green chemistry, as it can lead to the easy separation of these materials from the reaction medium.^23–27^ Chemical modification of SBA-15 can lead to the formation of versatile highly-efficient porous catalysts by additional active sites for interaction with the reactants.^28^ A catalytic application on SBA-15-based materials have been extensively studied in the literature.^29,30^ Safaei Ghomi *et al.* reported several of supported SBA-15 catalysts. Generally, in these reported works, the SBA-15 mesoporous silica was employed as a support metal species, and a highly dispersed metal catalyst was utilized in different multicomponent reactions,^31–33^ for example, they prepared functionalized-SBA-15 using a tetradentate ligand to construct a new catalyst. They investigated the efficiency of this catalyst in the synthesis of 2-azapyrrolizidine alkaloids, which have different biological properties.^34^ In 2019, by grafting the nickel Schiff base complex on the SBA-15 surface, Noroozi *et al.* prepared a nano-catalyst called Ni(ii)-Schiff base/SBA-15. It was an eco-friendly, and effective catalyst for the synthesis of 2-amino-4-aryl-5-oxo-4H,5H-pyrano[3,2-*c*]chromene-3-carbonitriles derivatives through a tandem reaction.^35^ In another example, in 2021, research on the synthesis of dihydropyranopyrazole compounds by using a guanidine functionalized SBA-15/Fe_3_O_4_ mesoporous nanocomposite was performed. In this research, synthesis efficiency of dihydropyranopyrazole derivatives by employing a guanidine functionalized SBA-15/Fe_3_O_4_, SBA-15/Fe_3_O_4_, and Fe_3_O_4_ reported 95%, 71%, and negligible, respectively, which is a good indication of the ability of SBA-15 in catalytic applications.^13^

Alginic acid, also called algin, is generally extracted from cell-wall of brown seaweeds such as macroalgae, ascophyllum, and kelp has attracted so much attention due to its natural availability and non-toxicity.^36,37^ Since this natural linear polysaccharide is obtained from natural resources, it can be utilized for construction of green catalysis of organic reactions.^38^ Brilliant properties such as chemical versatility, renewability, hydrophilicity, cost-effectiveness, and biodegradability make alginic acid as an environmentally friendly organocatalyst.^39,40^ However, the use of this natural polymer as a catalyst may be associated with weaknesses such as low chemical and physical stability, relatively low chemical variability, and undesirable catalytic efficiency that limit its catalytic application. Modification of alginic acid and adding it to heterogeneous systems seems to be a suitable solution to overcome these limitations and obtain a heterogeneous catalyst with high catalytic capacity.^38^

Herein, we introduce a new multifunctional catalytic system for the synthesis of pyrazolopyridine derivatives which mainly composed of SBA-15 as a mesoporous component, iron oxide MNPs as magnetic part, and modified alginic acid as natural polymer. In the fabrication of this novel mesoporous nanocomposite, formulated as “Alg@SBA-15/Fe_3_O_4_”, first SBA-15 was magnetized with Fe_3_O_4_ MNPs, then magnetized SBA-15 was modified with APTES to obtain SBA-15/Fe_3_O_4_–NH_2_. An important step in the preparation of the final composite is the conversion of alginic acid to methyl-esterified alginic acid *via* chemical esterification reaction. After the preparation of methyl-esterified alginic acid, in the final stage, the SBA-15/Fe_3_O_4_–NH_2_ formed a covalent bond with methyl-esterified alginic acid through nucleophilic substitution reaction. This inorganic–organic mesoporous nanocomposite has exhibited unique physicochemical characteristics which originate from the combination of its components. The outstanding characteristic properties of the fabricated heterogeneous catalytic system, for instance its porosity, average size, and thermal stability were carefully examined utilizing various analyses. Afterward, the ability of this catalyst in the organic synthesis was carefully investigated. In summary, it was found that using the Alg@SBA-15/Fe_3_O_4_ catalytic system at room temperature can lead to the synthesis of various high-yield pyrazolopyridine derivatives in short times. Also, the catalytic reusability of Alg@SBA-15/Fe_3_O_4_ was investigated for five successive runs, which showed that less than 10% of its catalytic capacity decreases after consecutive times compared to the first time. The easy separation of Alg@SBA-15/Fe_3_O_4_ catalytic system from the reaction medium stemmed from its magnetic property, is an effective step toward green chemistry. Overall, because of its cost-effectiveness and efficiency, the presented nanocatalyst is suggested for large-scale synthesis.

## 2. Results and discussion

### 2.1. Preparation of Alg@SBA-15/Fe_3_O_4_ nanocomposites

**Fig. 1:**
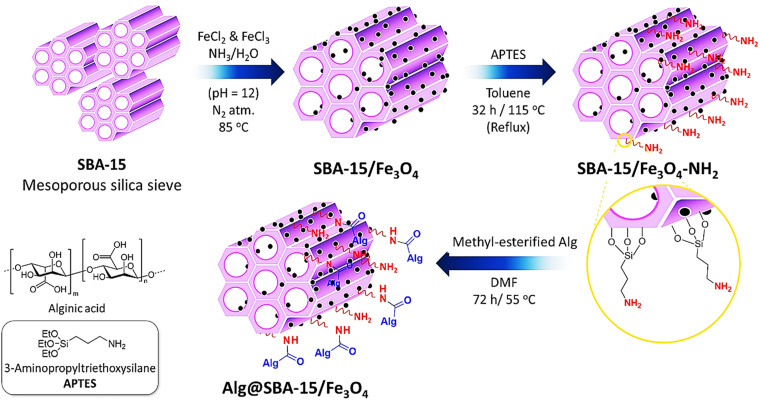
Schematic preparation route of Alg@SBA-15/Fe_3_O_4_ catalytic system.

To prepare Alg@SBA-15/Fe_3_O_4_ nanocomposite, several steps were performed which are briefly described in this section. After preparing SBA-15, the *in situ* co-precipitation method was used to magnetize the SBA-15, resulting in SBA-15/Fe_3_O_4_. Then, the modification of the magnetic SBA-15 surface was performed with APTES. The reason for using APTES in this study was that the surface of SBA-15 obtained the appropriate functional groups to bind to methyl-esterified alginic acid. Eventually, to prepare the Alg@SBA-15/Fe_3_O_4_ nanocomposites, the methyl-esterified alginic acid was attached to SBA-15/Fe_3_O_4_–NH_2_ through an amide bond (Fig. 1).

### 2.2. Characterization of Alg@SBA-15/Fe_3_O_4_ nanocomposites

#### 2.2.1. FTIR spectroscopy

**Fig. 2:**
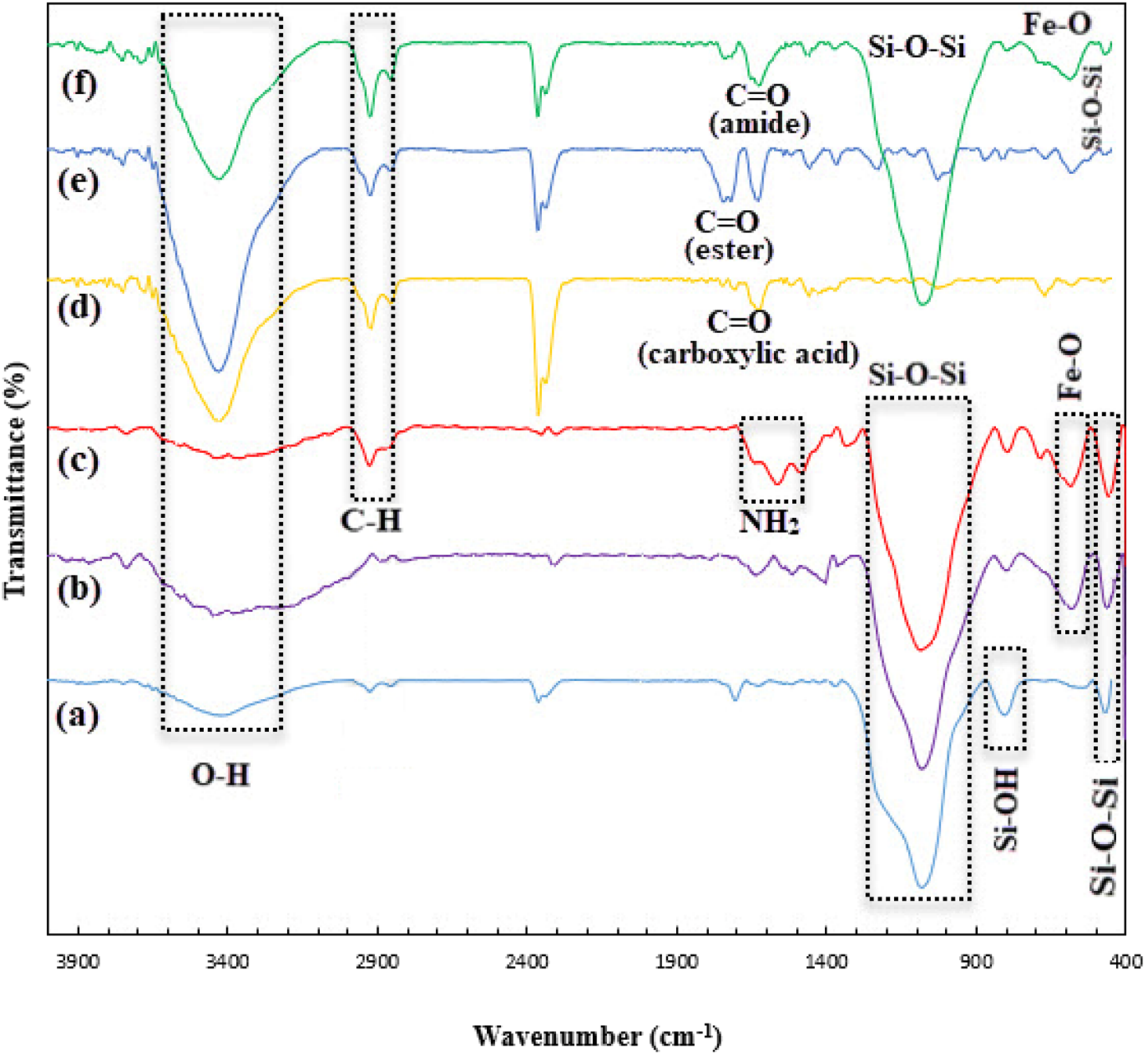
FTIR spectra of (a) SBA-15, (b) SBA-15/Fe_3_O_4_, (c) SBA-15/Fe_3_O_4_–NH_2_, (d) alginic acid, (e) methyl-esterified alginic acid, and (f) Alg@SBA-15/Fe_3_O_4_.

To investigate the existence of functional groups in prepared samples during the preparation of the final nanocomposite, FTIR analysis was utilized. The silica network has characteristic absorption bands at about 463 cm^−1^ (rocking bending vibration of Si–O–Si), about 804-960 cm^−1^ (stretching vibration of Si–OH), about 1084 cm^−1^ (symmetric stretching vibration of Si–O–Si), and about 3445 cm^−1^ (stretching vibration of OH groups) (Fig. 2a).^26,41,42^ Almost in the same areas of the SBA-15 peaks, there are peaks in the FTIR spectrum of SBA-15/Fe_3_O_4_, SBA-15/Fe_3_O_4_–NH_2_, and Alg@SBA-15/Fe_3_O_4_ (Fig. 2b, c and f), it can be a step towards proving the claim that these compounds contain SBA-15 in their structure. In the spectrum of SBA-15/Fe_3_O_4_ and Alg@SBA-15/Fe_3_O_4_, an absorption appeared at about 577 cm^−1^ associated to Fe–O vibration (Fig. 2b and f).^43,44^ After the prepared SBA-15/Fe_3_O_4_ was functionalized with APTES, two peaks emerged in its FTIR curve. One of them appeared in 1558 cm^−1^ related to the bending vibration of NH_2_, and the other one appeared in 2926 cm^−1^, which is related to the stretching vibrations of the C–H bond, which originate from the propyl chain of APTES (Fig. 2c).^13^ As can be seen in the FTIR curve of alginic acid (Fig. 2d), the peak of the carbonyl of the carboxylic acid functional group of alginic acid has observed at 1624 cm^−1^, while the carbonyl peak of the ester of methyl-esterified alginic acid appeared at 1720 cm^−1^ (Fig. 2e),^45^ this can confirm successful chemical esterification on alginic acid. The FTIR curve of the Alg@SBA-15/Fe_3_O_4_ (Fig. 2f) shows other peaks, the most important of them is the C=O peak at about 1624 cm^−1^, which probably belongs to the amide functional group. This peak is important in that it can be a reason for the creation of amide bonds between SBA-15/Fe_3_O_4_–NH_2_ and methyl-esterified alginic acid.

#### 2.2.2. XRD analysis

**Fig. 3:**
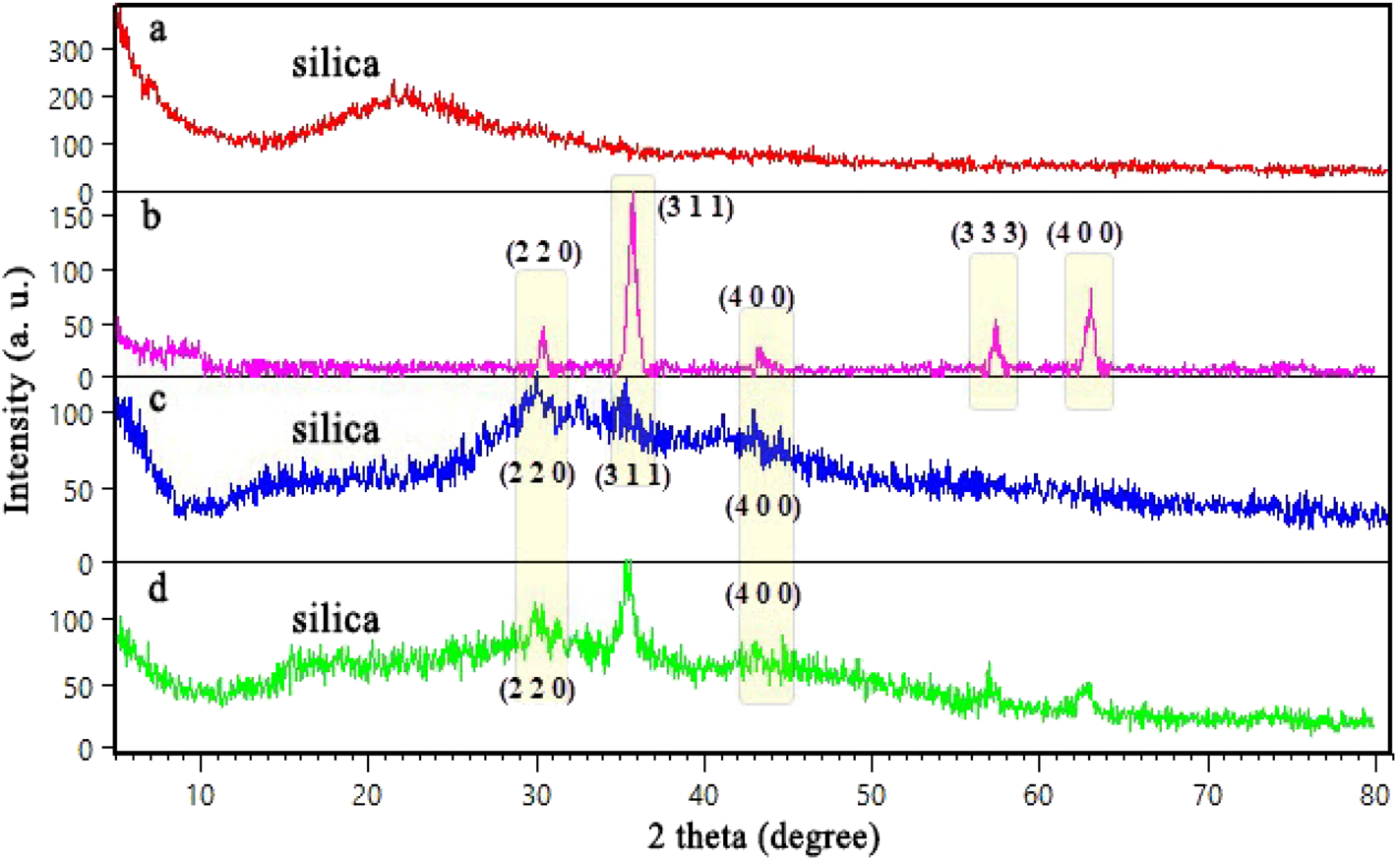
XRD patterns of (a) SBA-15, (b) Fe_3_O_4_ MNPs, (c) SBA-15/Fe_3_O_4_, (d) Alg@SBA-15/Fe_3_O_4_.

To characterize the crystalline structure of the samples, X-ray diffraction (XRD) analysis was carried out. The diffractograms of prepared Fe_3_O_4_ (Fig. 3b) shows characteristic diffraction peaks at 2*θ* = 30.43°, 35.86°, 43.47°, 57.38°, 63.19° were indexed to the (2 2 0), (3 1 1), (4 0 0), (3 3 3), (4 4 0) miller indices of cubic Fe_3_O_4_ with Card No. JCPDS 01-075-1609.^46^ According to Fig. 3a, the diffractograms of SBA-15 shows a broad distinctive peak in the range of 2*θ*: 20°–30° attributed to the amorphous SiO_2_ character.^47,48^ The XRD pattern of SBA-15/Fe_3_O_4_ shows peaks at 2*θ* = 30.35°(2 2 0), 35.98°(3 1 1), 43.56°(4 0 0), 57.70°(3 3 3) (Fig. 3b) that are also present in the diffractograms of the prepared Fe_3_O_4_ nanoparticles. This can be a reason to claim that SBA-15 has been magnetized with Fe_3_O_4_ nanoparticles. Similar to the XRD pattern of SBA-15, the XRD patterns of SBA-15/Fe_3_O_4_ and Alg@SBA-15/Fe_3_O_4_ (Fig. 3d) show a relatively broad peak at 2*θ*: 20°–30°, with less intensity in compared to unmodified SBA-15. It is worth mentioning that the lower intensity of the broad peak at 2*θ*: 20°–30° in Alg@SBA-15/Fe_3_O_4_ than its counterpart in SBA-15 may be due to the fact that SBA-15 was modified three times. Moreover, characteristic peaks of Fe_3_O_4_ MNPs were also observed at 2*θ*: 30.38°, 35.74°, 43.40°, 58°, 63.22°. It can be concluded that fabrication of Fe_3_O_4_ nanoparticles on SBA-15 substrate even with modifications has improved its crystallinity.

#### 2.2.3. TGA analysis

**Fig. 4:**
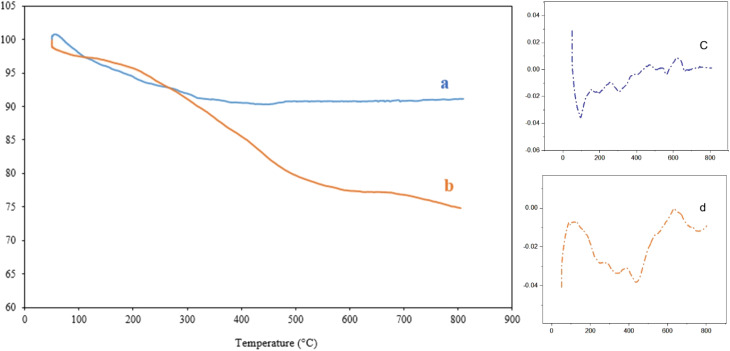
TGA/DTG curves of (a and c) SBA-15/Fe_3_O_4_, and (b and d) Alg@SBA-15/Fe_3_O_4_ nanocomposite systems.

The thermogravimetric analysis (TGA) curve of the SBA-15/Fe_3_O_4_ shows a steady weight loss in the range of 50 °C–800 °C with a mild slope (Fig. 4). The remarkable thermal stability of SBA-15/Fe_3_O_4_ is proven by the fact that only about 9% of its weight percentage is reduced up to 800 °C. This reduction is attributed to of vaporization of adsorbed water and moisture in the sample's cavities, as well as dehydrogenation or dehydroxylation of its surface.^13^ According to the TGA curve of Alg@SBA-15/Fe_3_O_4_, which is shown in Fig. 4, with increasing the sample temperature up to 800 °C, the mass percentage of Alg@SBA-15/Fe_3_O_4_ decreases with a gentle slope. More precisely, as the temperature increases from 50 °C to 160 °C, about 3% of the weight percentage of Alg@SBA-15/Fe_3_O_4_ decreases. This reduction is due to the vaporization of water and moisture absorbed inside the cavities of the nanocomposite and on its surface. As the temperature increases to 550 °C, the weight percentage of the sample decreases by about 18.5% in comparison with the weight percentage of the sample at 160 °C. This reduction may be because of the separation and thermal decomposition of the organic constituents of Alg@SBA-15/Fe_3_O_4_ (alkyl chain and alginic acid) attached to SBA-15 with covalent bonding. By increasing the temperature to 800 °C, the weight percentage of Alg@SBA-15/Fe_3_O_4_ decreases by more than 3% compared to the mass percentage of the same substance at 550 °C. This reduction might be related to the condensation of the silane groups of Alg@SBA-15/Fe_3_O_4_. After heating the nanocomposite to a temperature of 800 °C, its remaining weight was about 74.8%.^13^

#### 2.2.4. VSM analysis

**Fig. 5:**
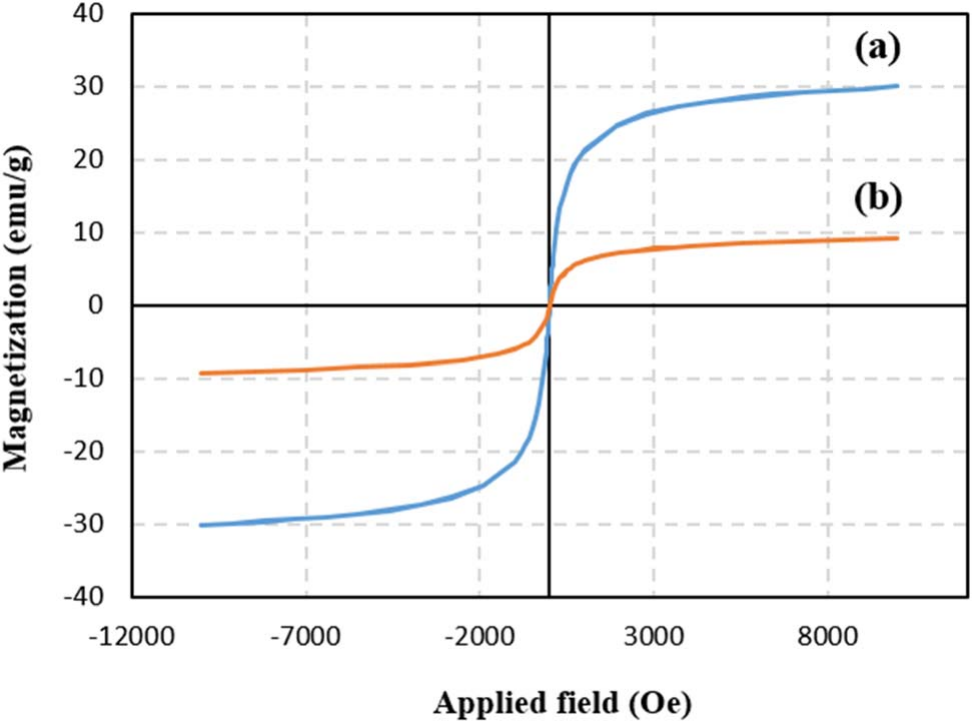
The magnetization curves of (a) SBA-15/Fe_3_O_4_, (b) Alg@SBA-15/Fe_3_O_4_.

Vibrating-sample magnetometer (VSM) was employed as a common technique for assessing magnetic properties of the materials at ambient temperature. As is observed in the magnetization curve of the Alg@SBA-15/Fe_3_O_4_, the absence of hysteresis loop, coercivity (Hc) and remanent magnetization (Mr) at room temperature indicate that the fabricated nanocomposite exhibited superparamagnetic (SPM) behavior. The magnetization saturation (Ms) of Alg@SBA-15/Fe_3_O_4_ is about 10 emu g^−1^ is less than the Ms value of SBA-15/Fe_3_O_4_ (Fig. 5), which seems to be due to the fact that a large part of Alg@SBA-15/Fe_3_O_4_ was composed of non-magnetic components.^13^ One of the significant properties of the fabricated catalyst in this study is its paramagnetic property. This feature allows Alg@SBA-15/Fe_3_O_4_ to be recycled magnetically and reused while maintaining performance.

#### 2.2.5. Electron microscopy and EDX

**Fig. 6:**
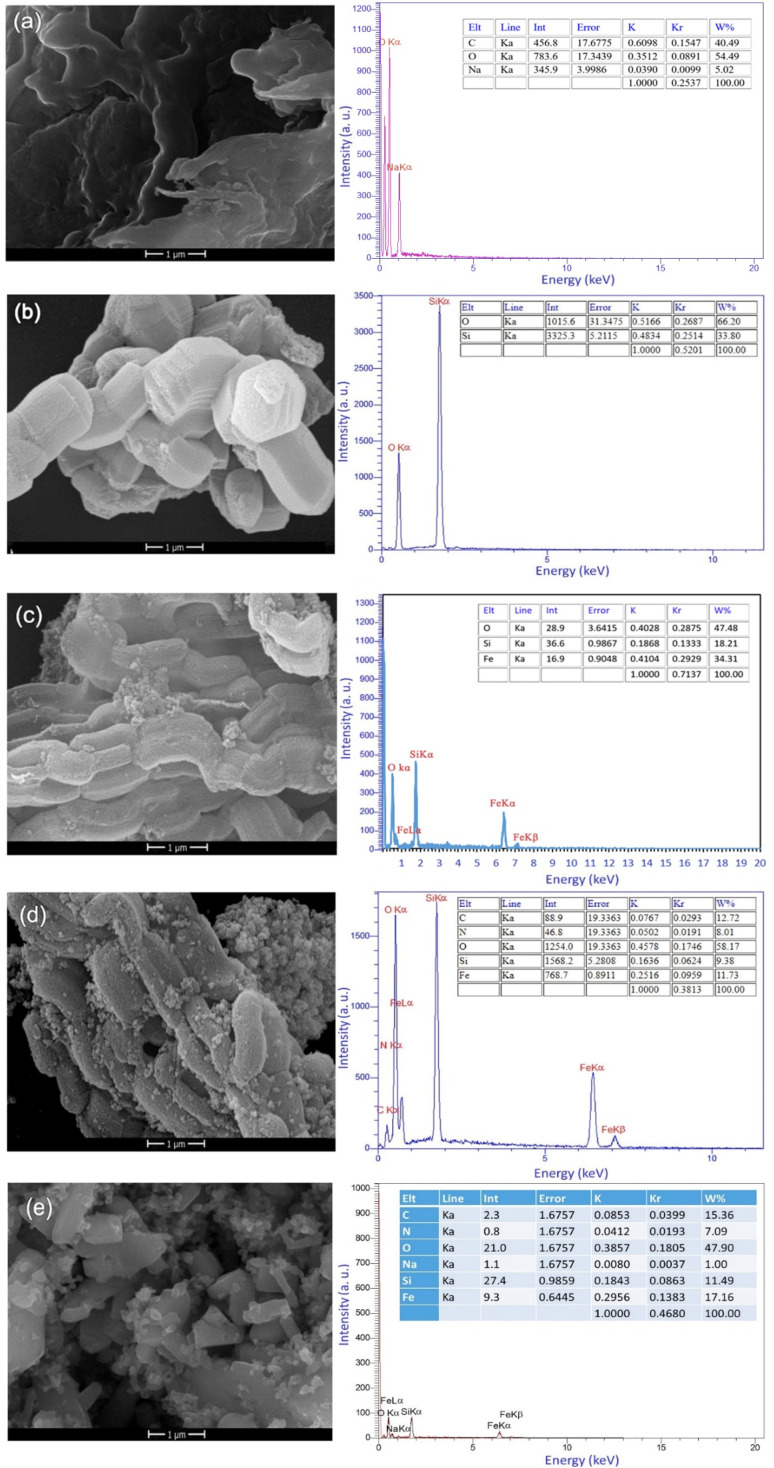
FESEM images and EDX spectra and tabulated data of (a) alginic acid, (b) SBA-15, (c) SBA-15/Fe_3_O_4_ (d) SBA-15/Fe_3_O_4_–NH_2_, and (e) Alg@SBA-15/Fe_3_O_4_.

FESEM was employed to study morphologies and structure of materials and prepared samples. Fig. 6a–e showed the FESEM images of the Alg, SBA-15, SBA-15/Fe_3_O_4_ and SBA-15/Fe_3_O_4_–NH_2_, and Alg@SBA-15/Fe_3_O_4_ in same scales (1 μm). Fig. 6a shows the morphology of alginic acid, and cylindrical shape of SBA-15 obviously can be seen in Fig. 6b. In Fig. 6c which belongs to SBA-15/Fe_3_O_4_, Fe_3_O_4_ NPs with a spherical shape are found on the cylindrical mesopore of the SBA-15. By further functionalization of SBA-15/Fe_3_O_4_ with APTES, the mesoporous structure of SBA-15 support with cylindrical shape has been retained. But in preparing the final composite structure, *i.e.* Alg@SBA-15/Fe_3_O_4_, SBA-15 underwent changes that resulted in changes in the morphology of SBA-15 (Fig. 6e). The chemical composition of the prepared samples were determined using EDX spectroscopy (Fig. 6). The EDX spectrum of each sample was presented next to its FESEM image. In the EDX spectrum of alginic acid, the C, N, O and Na peaks were detected (Fig. 6a). As is shown in the EDX spectrum of SBA-15, O and Si are the elemental constituent of SBA-15. The presence of O, Si and Fe elements in the EDX spectrum of SBA-15/Fe_3_O_4_ confirmed its chemical composition, observing two Fe peaks are connected to the existence of Fe_3_O_4_ MNPs in the SBA-15/Fe_3_O_4_ composition (Fig. 6b and c). The emerging the N and C peaks in the SBA-15/Fe_3_O_4_–NH_2_'s EDX spectrum, along with O, Si, and Fe peaks verified the successful modification of the SBA-15/Fe_3_O_4_ by the N-contained organosilane reagent, *i.e.*, APTES (Si–(CH_2_)_3_–NH_2_) (Fig. 6d). Finally, the EDX result of the final nanocomposite, the Alg@SBA-15/Fe_3_O_4_, proves the presence of all predicted elements including. O, Si, Fe, C, N, and Na (Fig. 6e). The elemental composition of O, Si, Fe, C, N, Na is 47.90, 11.49, 17.16, 15.36, 7.09, and 1.00%, respectively.

#### 2.2.6. The N_2_ adsorption–desorption isotherm

**Fig. 7:**
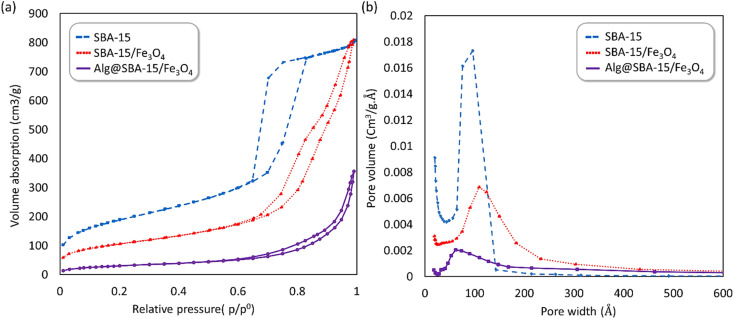
(a) N_2_ adsorption–desorption isotherms and (b) the pore size distribution curve of SBA-15 (blue), SBA-15/Fe_3_O_4_ (red) and Alg@SBA-15/Fe_3_O_4_ (purple).

The N_2_ adsorption–desorption isotherms of the SBA-15, SBA-15/Fe_3_O_4_, and Alg@SBA-15/Fe_3_O_4_ are shown in Fig. 7a. The type-IV curves that are typical of mesoporous compounds are seen in all three samples. The results of the surface area determined by the BET technique, the pore volume, and pore size (width) calculated by the BJH (Barrett–Joyner–Halenda) method are presented in Table 1. The surface area of the neat SBA-15 (687 m^2^ g^−1^) is more than the surface area of its magnetized compound, *i.e.*, SBA-15/Fe_3_O_4_ (384 m^2^ g^−1^) and Alg@SBA-15/Fe_3_O_4_ (about 113.4 m^2^ g^−1^). This result can be attributed to the blockage of a part of SBA-15 pores by Fe_3_O_4_ NPs incorporating on the pores of SBA-15 and its consequent modifications, which led to a reduction in accessible surface area for gas adsorption.^13,49^ In Table 1, the BJH pore size distributions of the prepared sample and compounds are shown. It can be seen that the SBA-15/Fe_3_O_4_ sample has a slightly larger pore size (diameter) and also has relatively broader pore size range than its precursor matrix.

**Table 1:** BET Surface area, pore volume, and pore size (diameter) of SBA-15, SBA-15/Fe_3_O_4_ and Alg@SBA-15/Fe_3_O_4_

Sample	Surface area^a^ (m^2^ g^−1^)	Pore volume^b^ (cm^3^ g^−1^)	Pore size^b^ (nm)
SBA-15	686.90	1.28	7.35
SBA-15/Fe_3_O_4_	383.91	1.26	12.64
Alg@SBA-15/Fe_3_O_4_	113.14	0.55	17.7

a The surface area obtained by BET technique.

b Pore volume and pore diameter measured by BJH technique.

### 2.3. Application of Alg@SBA-15/Fe3O4 composite in the synthesis of pyrazolopyridine derivatives

#### 2.3.1. Optimization of the catalyzed reaction conditions in the synthesis of pyrazolopyridine

To obtain the best catalytic performance of Alg@SBA-15/Fe_3_O_4_ nanocomposite for the synthesis of pyrazolopyridine derivatives, various experimental conditions were investigated^15^ in the four components reaction of ethyl acetoacetate (2.0 mmol), hydrazine hydrate (2.0 mmol), 3-nitrobezaldehyde (1.0 mmol) and ammonium acetate (1.0 mmol), the details of which are in Table 2. Firstly, the model multicomponent reaction was performed without Alg@SBA-15/Fe_3_O_4_ catalyst and solvent in two various temperatures, in which the product yield was inconsiderable (Table 2, entries 1 and 2). When the Alg@SBA-15/Fe_3_O_4_ nanocatalyst was added in the solvent free conditions, the reaction efficiency of the model reached approximately 65% (Table 2, entry 3). The further step, which can be seen in Table 2, entry 4, to examine the effect of the solvent, water was used as the solvent with 0.02 g of the Alg@SBA-15/Fe_3_O_4_ catalytic system at room temperature. In this case, the yield percentage of the reaction increased compared to the condition investigated in Table 2, entry 3. Among the obtained yields, the best was 97% obtained in EtOH media at room temperature (Table 2, entry 5). Even though the 97% efficiency was also obtained in Table 2, entry 9, but we prefer Table 2, entry 5 because less catalyst was used. Besides, the reaction condition and solvent, different amounts of Alg@SBA-15/Fe_3_O_4_ were investigated shown in Table 2, entries 7 to 9. The yield of the model multicomponent reaction was also examined under other different conditions which are shown in Table 2, entries 10 and 11. Also, the catalytic ability of a mixture of Alg and SBA-15/Fe_3_O_4_ and a mixture of methyl-esterified Alg and SBA-15/Fe_3_O_4_ were investigated, which showed yields of 91% and 90%, respectively (Table 2, entries 13 and 14). Although these efficiencies are high and close to the catalytic efficiency shown by the Alg@SBA-15/Fe_3_O_4_ catalytic system (Table 2, entry 5), an important issue in the performance of catalysts is that catalytic systems must be stable in the reaction medium. The Alg@SBA-15/Fe_3_O_4_ catalytic system, in addition to having high catalytic efficiency, has significant stability in the reaction medium.

**Table 2:** Optimizing the multicomponent reaction conditions in the synthesis of pyrazolopyridine derivatives

Entry	Catalyst	Catalyst loading (g)	Solvent	Temp. (°C)	Yield^a^ (%)
1	—	—	—	r.t.	Trace
2	—	—	—	80	Trace
3	Alg@SBA-15/Fe_3_O_4_	0.02	—	r.t.	<65
4	Alg@SBA-15/Fe_3_O_4_	0.02	H_2_O	r.t.	78
5	**Alg@SBA-15/Fe**_**3**_ **O**_**4**_	**0.02**	**EtOH**	**r.t.**	**97**
6	Alg@SBA-15/Fe_3_O_4_	0.02	EtOH/H_2_O (1 : 1)	r.t.	89
7	Alg@SBA-15/Fe_3_O_4_	0.015	EtOH	r.t.	94
8	Alg@SBA-15/Fe_3_O_4_	0.010	EtOH	r.t.	92
9	Alg@SBA-15/Fe_3_O_4_	0.025	EtOH	r.t.	97
10	Alg	0.020	EtOH	r.t.	57
11	SBA-15/Fe_3_O_4_	0.020	EtOH	r.t.	58
12	Methyl-esterified Alg	0.020	EtOH	r.t.	48
13	Mixture of Alg and SBA-15/Fe_3_O_4_	0.020	EtOH	r.t.	**91**
14	Mixture of methyl-esterified Alg and SBA-15/Fe_3_O_4_	0.020	EtOH	r.t.	**90**

a Multicomponent reaction conditions: ethyl acetoacetate (2 mmol), hydrazine hydrate (2 mmol), 3-nitrobezaldehyde (1 mmol) and ammonium acetate (1 mmol), catalyst (10–25 mg). The yields relate to the isolated product.

#### 2.3.2. Catalyzed synthesis of the pyrazolopyridine derivatives

**Scheme 1:**
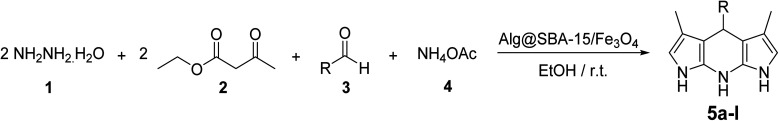
Synthesis of pyrazolopyridine derivatives (5a–l) in the presence of Alg@SBA-15/Fe_3_O_4_ nanocatalyst.

To assess the catalytic efficacy of the fabricated Alg@SBA-15/Fe_3_O_4_ composite, the organic synthesis of pyrazolopyridine derivatives was investigated (Scheme 1), and the results are summarized in Table 3. Interestingly, a wide range of high-yield pyrazolopyridine derivatives were obtained with using the Alg@SBA-15/Fe_3_O_4_ catalyst in 20 to 30 minutes.

**Table 3:** Synthesis yields of different pyrazolopyridine derivatives by using Alg@SBA-15/Fe_3_O_4_ catalyst under the optimized conditions

Entry	Aldehyde	Product	Time (min)	Yield^a^ (%)	Mp (°C)	
					Found	Reported
1	Benzaldehyde	5a	25	95	240–243	240–241 (ref. 8)
2	3-NO_2_ benzaldehyde	5b	20	97	287–290	285–287 (ref. 8)
3	2,4-Cl_2_ benzaldehyde	5c	20	97	307–310	308 (ref. 8)
4	4-Cl benzaldehyde	5d	20	93	257–259	255–257 (ref. 8)
5	4-Me benzaldehyde	5e	25	97	246–248	244–246 (ref. 8)
6	4-OMe benzaldehyde	5f	30	92	184–186	185–186 (ref. 50)
7	3-Me benzaldehyde	5g	30	90	279–280	280–282 (ref. 51)
8	2-Cl benzaldehyde	5h	30	93	220–223	219–221 (ref. 52)
9	2-Me benzaldehyde	5i	25	91	290–293	290–292 (ref. 53)
10	4-F benzaldehyde	5j	20	92	258–260	259–261 (ref. 8)
11	2-OH, 3-OMe benzaldehyde	5k	30	91	202–204	200–202 (ref. 54)
12	3,4,5-(OMe)_3_ benzaldehyde	5l	25	93	249–251	248–250 (ref. 51)
13	4-CH(Me)_2_ benzaldehyde	5m	30	91	281–284	280–282 (ref. 51)

a The yields relate to the isolated product.

#### 2.3.3. Suggested mechanism

**Fig. 8:**
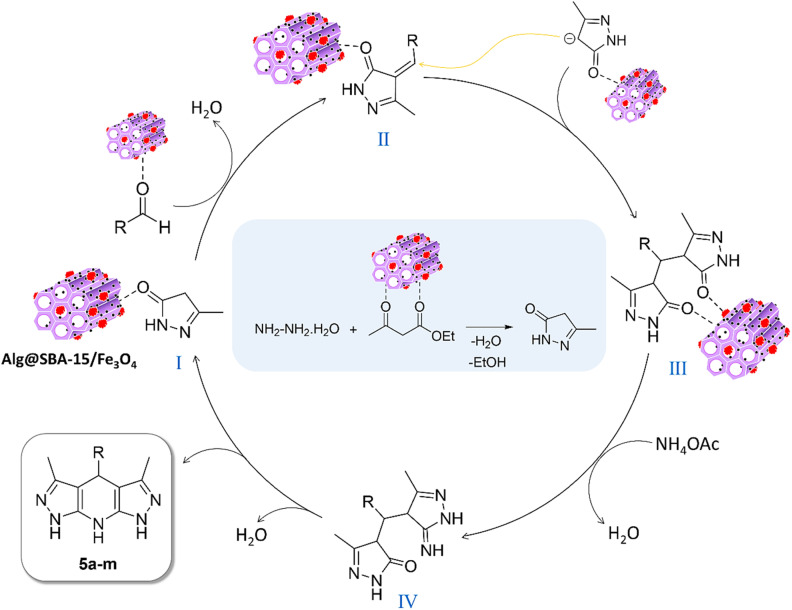
A plausible mechanism for catalyzing the synthesis reaction of pyrazolopyridine derivatives by Alg@SBA-15/Fe_3_O_4_.

The most likely assisted mechanism to synthesis of the different pyrazolopyridine derivatives with Alg@SBA-15/Fe_3_O_4_ catalyst is a four-steps is shown in Fig. 8. The Alg@SBA-15/Fe_3_O_4_ mesoporous nanocomposite by having abundant hydroxyl groups, amides and to some extent carboxylates, Lewis acid site (Fe^3+^ in Fe_3_O_4_), and also because of its physical properties such as high porous structure as well as great surface area, can play a critical role in activation of reactants in all steps of this multicomponent reaction as demonstrated in Fig. 8.^13^ Initially, Alg@SBA-15/Fe_3_O_4_ activates the carbonyl ethyl acetoacetate group by forming a hydrogen bond and also by interacting with Lewis acid sites followed by nucleophilic attack of hydrazine on them, which is accompanied by a ring–forming reaction, intermediate I, a pyrazolone ring, is formed. Next, *via* the Knoevenagel condensation reaction between the activated aldehyde and the pyrazolone ring, intermediate II is formed. The further step, by performing the Michael addition reaction between the pyrazolone ring and intermediate II, intermediate III is formed. Then, intermediate IV is constructed by the nucleophilic attack of ammonia (from ammonium acetate) on intermediate III with the aid of the Alg@SBA-15/Fe_3_O_4_ catalyst. In the last step, through intramolecular ring formation of intermediate IV, which leads to the removal of water from it, as well as product totomerism, the final product of pyrazolopyridine is synthesized.

#### 2.3.4. Catalyst recyclability and stability

**Fig. 9:**
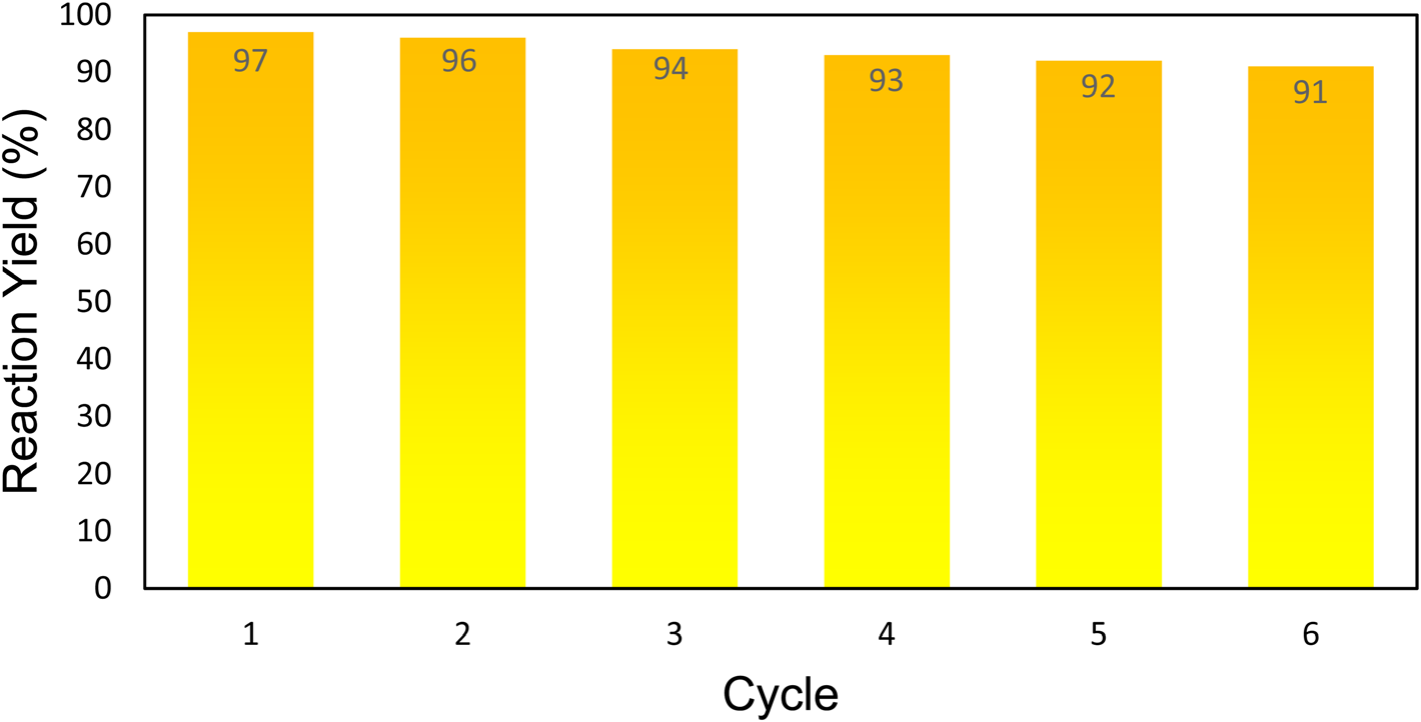
Recycling diagram of the Alg@SBA-15/Fe_3_O_4_ catalyst in the synthesis of 5b.

**Fig. 10:**
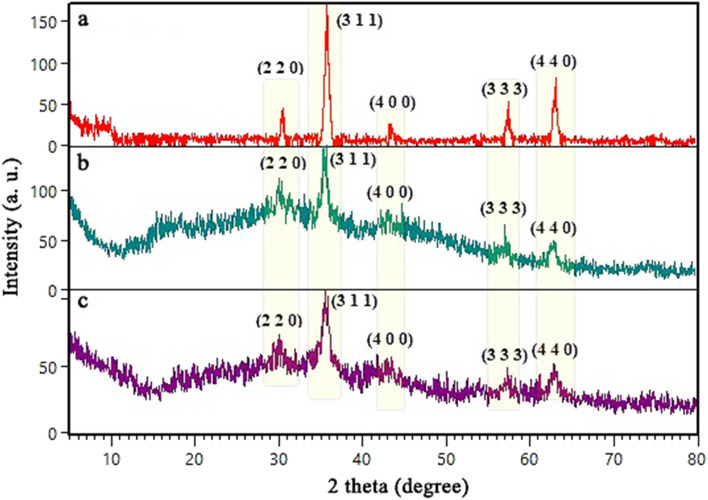
The XRD pattern of (a) Fe_3_O_4_ MNPs, (b) the fresh Alg@SBA-15/Fe_3_O_4_ catalyst (c) after five times reusing in the reaction.

In this study, the reusability of the prepared Alg@SBA-15/Fe_3_O_4_ catalytic system in the synthesis of pyrazolopyridine derivatives was evaluated, since it is an important issue in green chemistry.^55–57^ After the reaction was completed, the Alg@SBA-15/Fe_3_O_4_ catalyst was isolated from the reaction medium by using an external magnet and then diluted with ethanol and dried in an oven at 55 °C for 6 h to prepare for the subsequent catalytic run. For following runs, a fixed amount of the recovered Alg@SBA-15/Fe_3_O_4_ catalyst was utilized. According to Fig. 9, after five consecutive uses of Alg@SBA-15/Fe_3_O_4_ catalyst in the synthesis of pyrazolopyridine derivatives, less than 9% of its catalytic power was reduced compared to the first use in the same reaction. As a result, the Alg@SBA-15/Fe_3_O_4_ catalyst can be used for five runs without considerable reduction in its catalytic activity. Furthermore, the XRD analysis of the recycled catalyst (Fig. 10) was also performed and compared with diffractograms of the Fe_3_O_4_ MNPs and the fresh Alg@SBA-15/Fe_3_O_4_ catalyst to determine the structural and mechanical stability of the fabricated catalyst. The results indicate that the structure of the Alg@SBA-15/Fe_3_O_4_ has remained unchanged after reuses in the reaction compared to fresh catalysts and all of the indicative peaks of the nanocomposite appeared in XRD pattern of recycled one.

## 3. Experimental section

### 3.1. Materials and equipment

All used chemicals in present work, were bought from Merck and Sigma-Aldrich companies. In order to show the correct construction of the Alg@SBA-15/Fe_3_O_4_ catalyst and its characteristics, several analyses were used. A Shimadzu IR-470 spectrometer was used to get Fourier-transform infrared (FT-IR) spectra of the samples. Energy dispersive X-ray analysis (EDX) of the neat materials and prepared samples was performed employing a Numerix DXP-X10P and TESCAN, MIRA II. The morphology and surface property of neat materials and prepared compounds were examined with the help of images obtained from FESEM (model KYKY-EM8000). By employing a Lakeshore 7407, the magnetic behavior of the samples was studied at ambient temperature. By utilizing an STA504 device, thermal stability of compounds was assessed by TGA. The produced samples' X-ray diffraction (XRD) pattern was achieved utilizing an X-ray diffractometer (JEOL JDX-8030 (20 mA, 30 kV)). The N_2_ adsorption–desorption isotherm of this study was also obtained using Micromeritics ASAP 2010. Also, a, KQ-250 DE (40 kHz) device was utilized as an ultrasound cleaning bath.

### 3.2. Preparation method

#### 3.2.1. Synthesis of SBA-15

Well-ordered SBA-15 was synthesized by hydrothermal method. For this, in a 250 mL flask, 3.5 mL of pluronic P123, 120.0 mL of distilled water and 20.0 mL of 37% hydrochloric acid were poured and then stirred at room temperature to mix thoroughly. Then, after the temperature of the reaction mixture fixed at 40 °C, the flask containing the components was stirred at 40 °C for 24 h. After this time, 8.5 mL of TEOS (tetraethyl orthosilicate) was dropped to the above solution while stirring vigorously. After the addition of TEOS, the resulting mixture was spun at 120 rpm for a day. Next, the reaction mixture was transferred to a teflon-lined autoclave reactor and heated at 150 °C for 24 h in an oven. After the synthesis process, the resulting mixture cooled to room temperature and the resulting sediment was separated by filtration. The resulting solid was rinsed with distilled water many times and dried overnight at 100 °C. The dried powder was calcination was performed by heating for 5 h at 500 °C to remove of surfactant and constructing the ordered channel of mesoporous SBA-15.

#### 3.2.2. Synthesis of SBA-15/Fe_3_O_4_

0.43 g of FeCl_2_·4H_2_O and 0.54 g of FeCl_3_·6H_2_O with 100.0 mL of distilled water were poured into a 250 mL flask and stirred for 40 minutes at room temperature. Then 0.5 g of white powder of SBA-15 which was synthesized in the previous step was added to the reaction mixture under N_2_ atmosphere and stirred for 30 min. After raising the reaction mixture temperature to 80 °C, 10.0 mL of 25% ammonia solution was dropped to the reaction mixture. The resulting brown mixture was stirred at 80 °C for one hour. Using an external magnet, SBA-15/Fe_3_O_4_ was isolated, and it was washed many times with distilled water. The resulting product was placed in an oven at 100 °C for several hours to dry.

#### 3.2.3. Synthesis of SBA-15/Fe_3_O_4_–NH_2_

1.0 g of SBA-15/Fe_3_O_4_ synthesized in the previous step was poured into 40.0 mL of toluene in a 100 mL flask. After that, an ultrasonic bath was used to thoroughly disperse the reaction mixture. After that, 2.0 mL of APTES was added and the temperature was increased to 120 °C, then it was stirred under reflux for 32 h. After these 32 h, the precipitate was washed several times with water and ethanol and placed in an oven at 60 °C for 12 h to dry.

#### 3.2.4. Preparation of methyl-esterified alginic acid

Methyl-esterified alginic acid was prepared *via* chemical esterification of alginic acid by using methanol in mildly acidic aqueous solution.^58^ 2.0 g of alginic acid was poured in a 500 mL flask containing 200.0 mL of methanol and 2.0 mL of sulfuric acid 98%. Then, the temperature of the reaction was increased to 60 °C and it was stirred for 72 h at the same temperature. After 72 h, the resulting powder was diluted with distilled water and acetone, then placed in an oven at 55 °C to dry.

#### 3.2.5. Synthesis of Alg@SBA-15/Fe_3_O_4_

0.7 g of synthesized SBA-15/Fe_3_O_4_–NH_2_ with 0.05 g of methyl-esterified alginic acid were poured in 10 mL of DMF solvent and was stirred for 72 h at 55 °C. The product was then gathered using a magnet and rinsed three times with distilled water and ethanol. Afterwards it was dried in an oven at 55 °C.

### 3.3. Procedure for the synthesis of pyrazolopyridine derivatives with the Alg@SBA-15/Fe_3_O_4_ catalytic system

Four reactants including; ethyl acetoacetate (2.0 mmol), hydrazine hydrate (2.0 mmol), aromatic aldehydes (1.0 mmol) and ammonium acetate (1.0 mmol) were mixed and reacted in the presence of 0.02 g of the Alg@SBA-15/Fe_3_O_4_ catalytic system in the presence of 2.0 mL EtOH at room temperature. TLC (Thin layer chromatography) technique was employed to investigate the reaction completion process. After the reaction was finished, the magnetic nanocatalyst was effortlessly removed from the reaction medium by an external magnet. In the final step, by adding water to the reaction mixture, pure products were got, filtered and washed with further water. Most of the time, additional purification was not required.

#### 3.3.1. Spectral data of two products

##### 3.3.1.1 3,5-Dimethyl-4-phenyl-1,4,7,8-tetrahydrodipyrazolo[3,4-*b*:4′,3′-*e*] pyridine (5a)

FT-IR (KBr) *ν*_max_: 3540, 3301, 2925, 1614, 1515, 1375 cm^−1^; ^1^H-NMR (300 MHz, DMSO-d_6_): *δ*_H_ (ppm) = 2.06 (6H, 2-CH_3_, singlet), 4.81 (1H, –CH, singlet), 7.14–7.20 (5H, H-aromatic, multi), 11.24–11.32 (3H, –NH, broad singlet); ^13^C NMR (75 MHz, DMSO-d_6_): *δ* (ppm) = 10.8, 33.1, 104.6, 125.8, 127.9, 128.1, 140.2, 143.7, 161.5. CHN Anal. Calcd for C_15_H_15_N_5_: C: 67.90, H: 5.70, N: 26.40 found C: 67.86, H: 5.67, N: 26.37, mass for C_15_H_15_N_5_: [M–H] 265.133 found 265.122.

##### 3.3.1.2 4-(4-Chlorophenyl)-3,5-dimethyl-1, 4,7,8-tetrahydrodipyrazolo[3,4-*b*:4′,3′-*e*] pyridine (5d)

FT-IR (KBr) *ν*_max_: 3100, 2854, 1612, 1526, 1368 cm^−1^;^1^H-NMR (300 MHz, DMSO-d_6_): *δ*_H_ (ppm) = 2.06 (6H, 2-CH_3_, singlet), 4.8 (1H, –CH, singlet), 7.12 (5H, H-aromatic, multi),7.24 (2H, H-aromatic, broad singlet), 11.35 (3H, –NH, broad singlet). ^13^C NMR (75.47 MHz, DMSO-d6): *δ*(ppm) = 10.7, 32.6, 104.3, 128.0, 129.8, 130.4, 140.1, 142.7, 161.4. CHN Anal. Calcd for C_15_H_14_ClN_5_: C: 60.10, H: 4.71, N: 23.36 found C: 60.08, H: 4.69, N: 23.35.

##### 3.3.1.3 4-(4-Fluorophenyl)-3,5-dimethyl-1,4,7,8-tetrahydrodipyrazolo[3,4-*b*:4′,3′-*e*]pyridine (5j)

FT-IR (KBr) *ν*_max_: 3165, 3067, 1602, 1511; ^1^HNMR (300.13 MHz, DMSO-d_6_): *δ*_H_ (ppm) = 2.07 (6H, 2-CH_3_, singlet), 4.81 (1H, –CH, singlet), 7.01–7.05 (2H, H-aromatic, multi), 7.08–7.13 (*m*, 2H, H-Aromatic, multi), 11.34 (3H, –NH, broad singlet); ^13^C NMR (75.47 MHz, DMSO-d_6_): *δ* = 10.8, 32.5, 104.6, 114.8, 129.6, 129.7, 139.8, 161.4,162.4; Anal. Calcd for C_15_H_14_FN_5_: C: 63.59, H: 4.98, N: 24.72 found C: 63.57, H: 5.01, N: 24.69; mass for C_15_H_14_FN_5_ [M–H] 283.12 found 283.11.

## 4. Conclusion

In this study, a novel mesoporous nanocomposite based on SBA-15 was fabricated in which SBA-15 was modified with Fe_3_O_4_ nanoparticles, APTES and methyl-esterified alginic acid. For the synthesis of pyrazolopyridine derivatives, the prepared nanocomposite demonstrated excellent catalytic performance. The corresponding products were synthesized in high yields without using a complicated work-up process. TGA result revealed that this nanocomposite is thermally stable, losing only around 25% of its weight up to 800 °C. The XRD analysis also confirmed that Alg@SBA-15/Fe_3_O_4_ has properly been prepared. Also, the results of other analyzes, including VSM, FESEM, BET and EDX, were consistent with the confirmation of the correct synthesis of Alg@SBA-15/Fe_3_O_4_. The catalytic system synthesized in this study can be considered efficient catalyst for the synthesis of pyrazolopyridine derivatives. One of the reasons for this is its superparamagnetic property, which was also confirmed by VSM analysis, which allows this catalyst to be easily separated from the medium and reused for several times. In the model reaction of this study for the synthesis of various derivatives of pyrazolopyridine, while a small amount of Alg@SBA-15/Fe_3_O_4_ (0.02 g) was used, high yield products were obtained in a short time.

## Conflicts of interest

The authors declare no conflict of interest.

## Supplementary Material

RA-013-D2RA07228A-s001
