# Supplementary material for: Facile synthesis of pyrazolopyridine pharmaceuticals under mild conditions using an algin-functionalized silica-based magnetic nanocatalyst (Alg@SBA-15/Fe3O4)
Source: RSC Adv. 2023 Apr 3;13(15):10367–78. doi: 10.1039/d2ra07228a (PMC10068431; doi:10.1039/d2ra07228a)
Supplement: RA-013-D2RA07228A-s001 [file RA-013-D2RA07228A-s001.pdf]

Electronic Supplementary Material (ESI) for RSC Advances.
This journal is © The Royal Society of Chemistry 2023

Supplementary Material

Facile Synthesis of Pyrazolopyridine Pharmaceuticals under Mild Conditions by an Algin-Functionalized Silica-Based Magnetic Nanocatalyst (Alg@SBA-15/Fe_3O_4)

*Fereshte Hassanzadeh-Afruzi, Zeinab Amiri-Khamakani, Mahdi Saeidirad, Reza Taheri-Ledari, Ali Maleki**

Catalysts and Organic Synthesis Research Laboratory, Department of Chemistry, Iran University of Science and Technology, Tehran 16846-13114, Iran.

**Corresponding author. E-mail: maleki@iust.ac.ir; Fax: +98-21-73021584; Tel: +98-21-73228313.*

Author's ORCIDs:
Fereshteh Hasanzadeh-Afruzi: https://orcid.org/0000-0003-0570-2506
Reza Taheri-Ledari: https://orcid.org/0000-0002-6511-9411
Ali Maleki: https://orcid.org/0000-0001-5490-3350

Table of Content

Content	Page
Comparison of the prepared Alg@SBA-15/Fe_3O_4 with other catalytic systems	**S1-S2**
Figure S1-S8-Copies of ^{1}H, ^{13}C NMR, and Mass spectra of compounds **5a**, **5d**, **5 j**	**S3-10**

Comparison of the prepared Alg@SBA-15/Fe_3O_4 with other catalytic systems

In order to better understand the catalytic performance and advantages of the Alg@SBA-15/Fe_3O_4 catalytic system in the synthesis of pyrazolopyridine derivatives, the necessary conditions and yield of fabricated catalytic system in the synthesis of 5b derivative was compared with a number of other catalysts that have synthesized the same product so far. According to Table 1S (see supplementary information), it seems that the method investigated in this study is appropriate approach for the pyrazolopyridine derivatives.

Table S1. Comparison of the catalytic activity of the Alg@SBA-15/Fe_3O_4 with several reported catalysts for the synthesis of pyrazolopyridine derivatives (product **5b**).

Entry	Catalyst	Catalyst loading	Conditions	Yield[a] (%)
1	$CuFe_2O_4$@HNTs nanocatalyst	0.05 g	EtOH, room temperature, 20 min	95[8]
2	pseudopolymeric magnetic nanoparticles	0.01 g	EtOH, room temperature, 120 min	45[43]
3	$FeNi_3$-ILs MNPs	0.002 g	EtOH, 45 min	84[44]
4	Nano-$CuCr_2O_4$	4 mol%	EtOH, 45 min, 25 °C	91[48]
5	Fe_3O_4@SiO_2-SO_3H NPs	0.004 g	EtOH, 25 °C, 15 min, 500 MW	94[49]
6	Alg@SBA-15/Fe_3O_4	0.02 g	20 min, EtOH, r.t.	97[a]

[a] *This work*

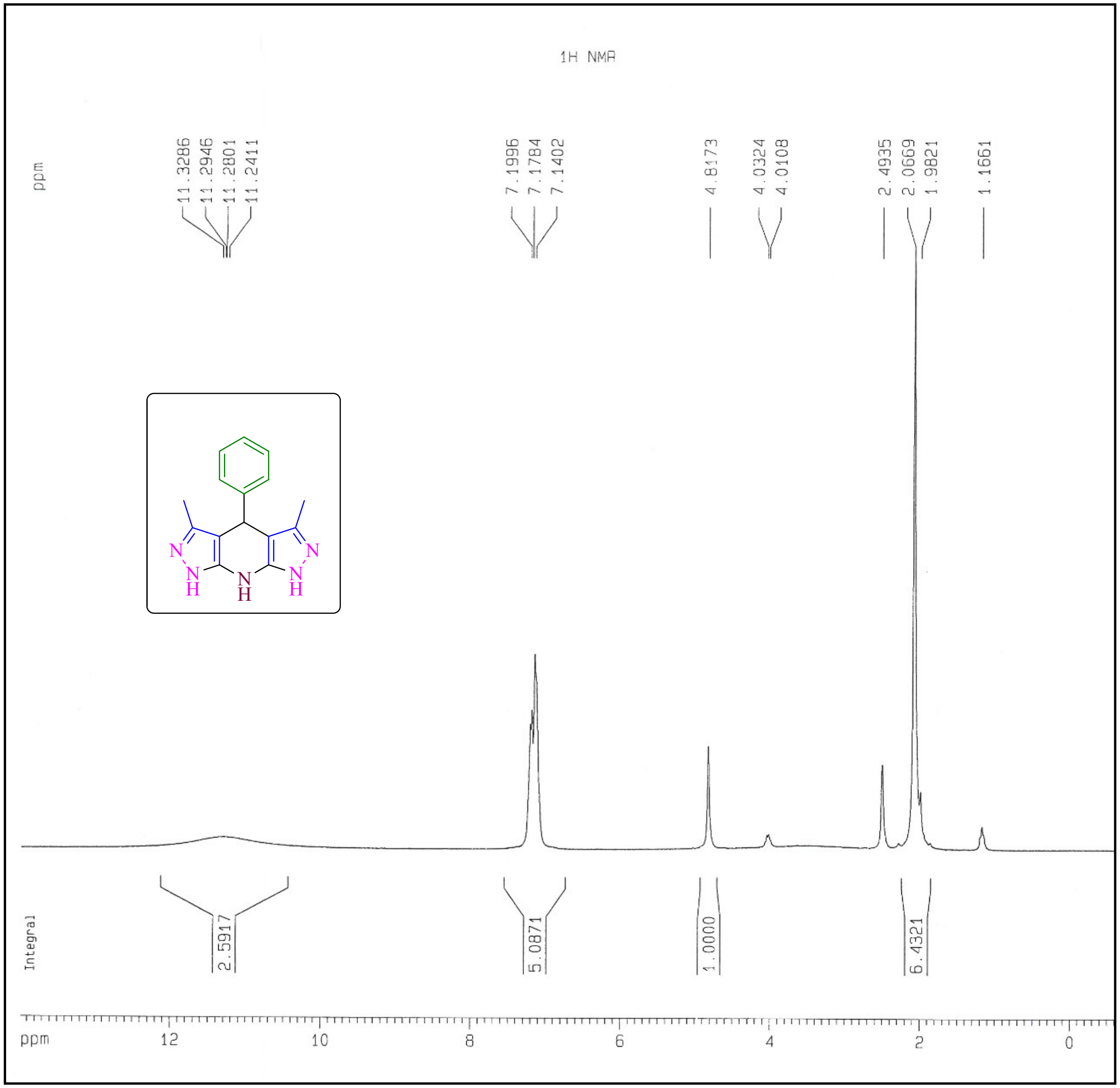

Figure S1. [13]H NMR spectrum of compound (**5a**)

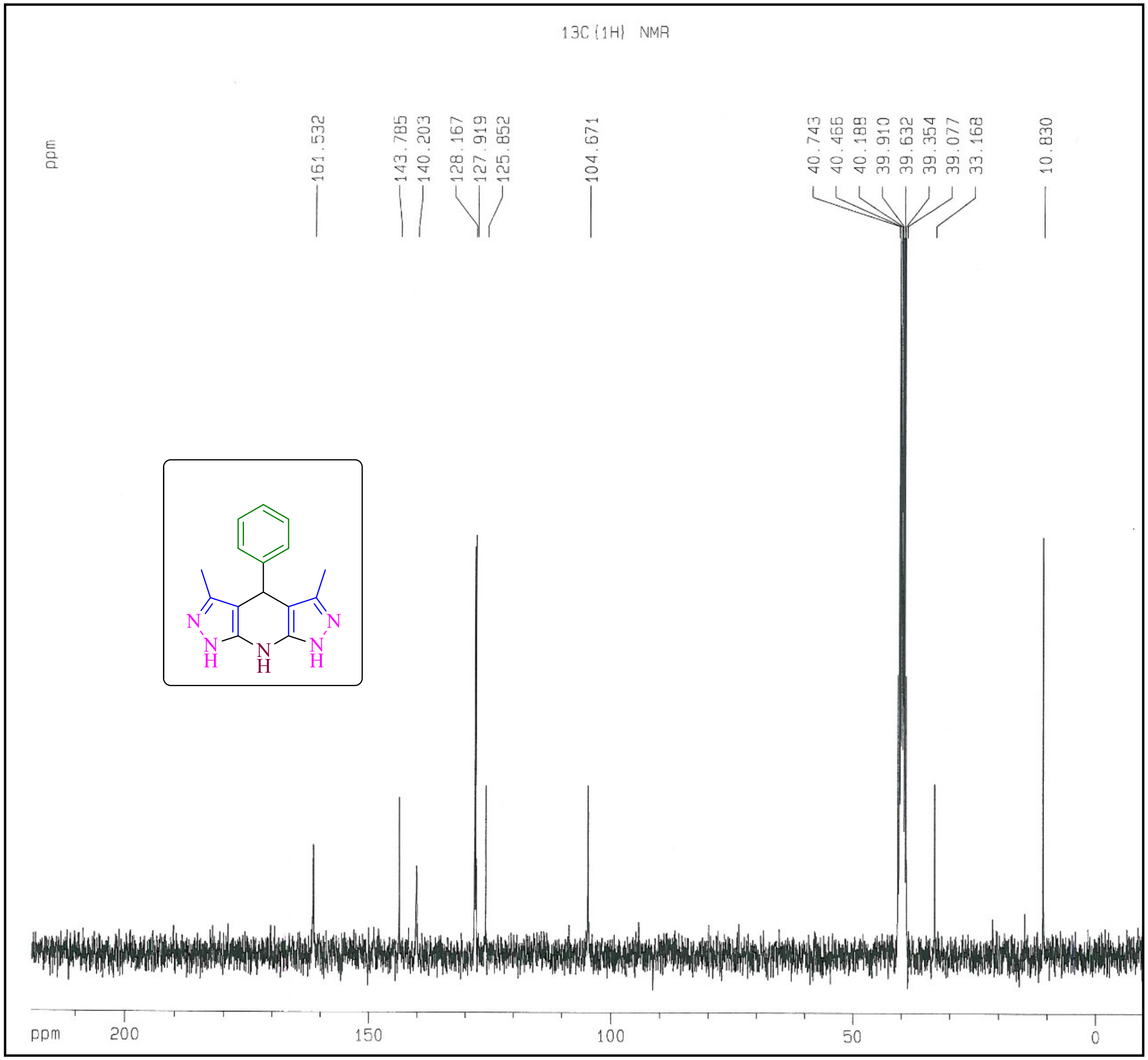

Figure S2. ^{13}C NMR spectrum of compound (**5a**)

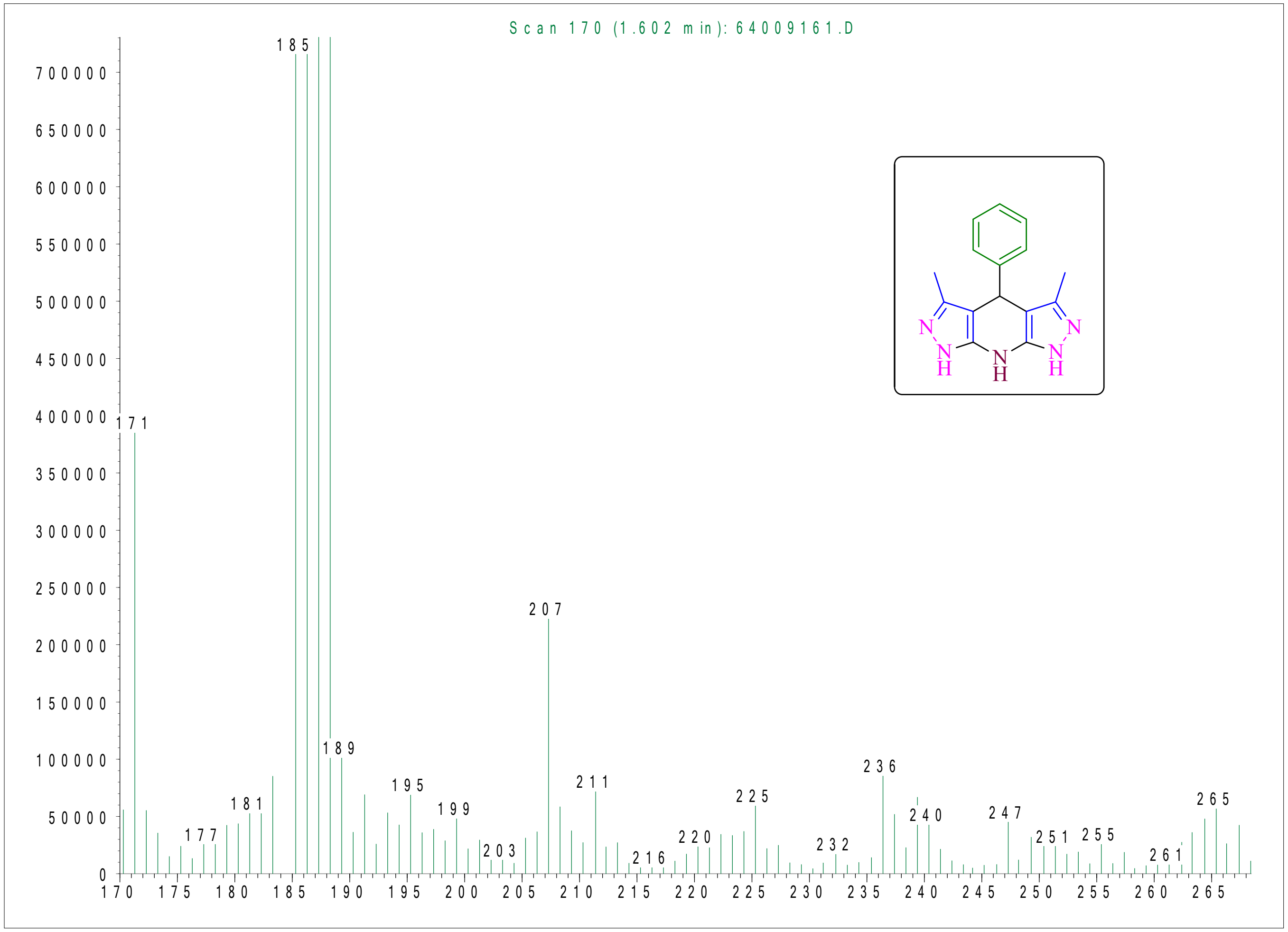

Figure S3. Mass spectrum of compound (**5a**)

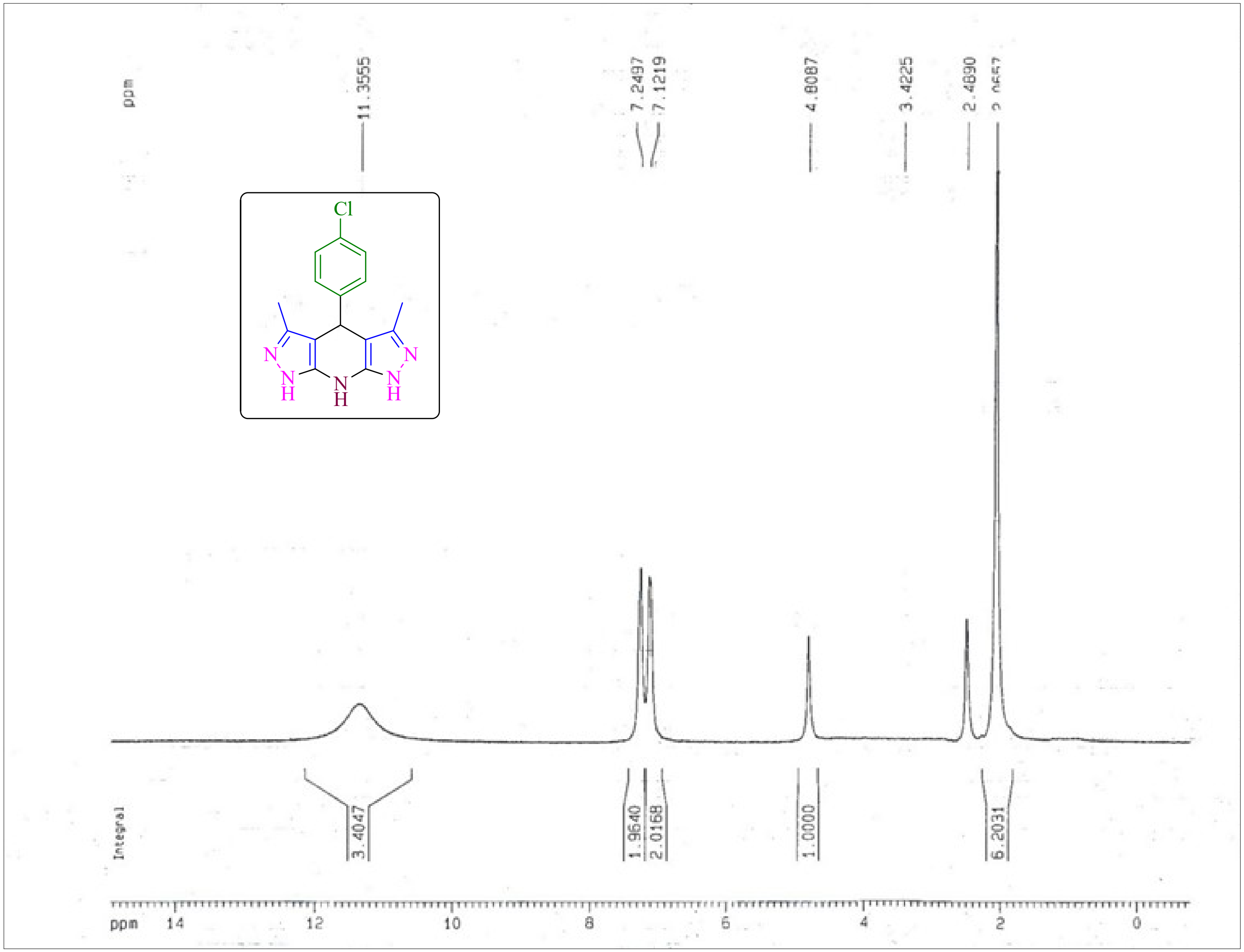

Figure S4. ^{13}H NMR spectrum of compound (**5d**)

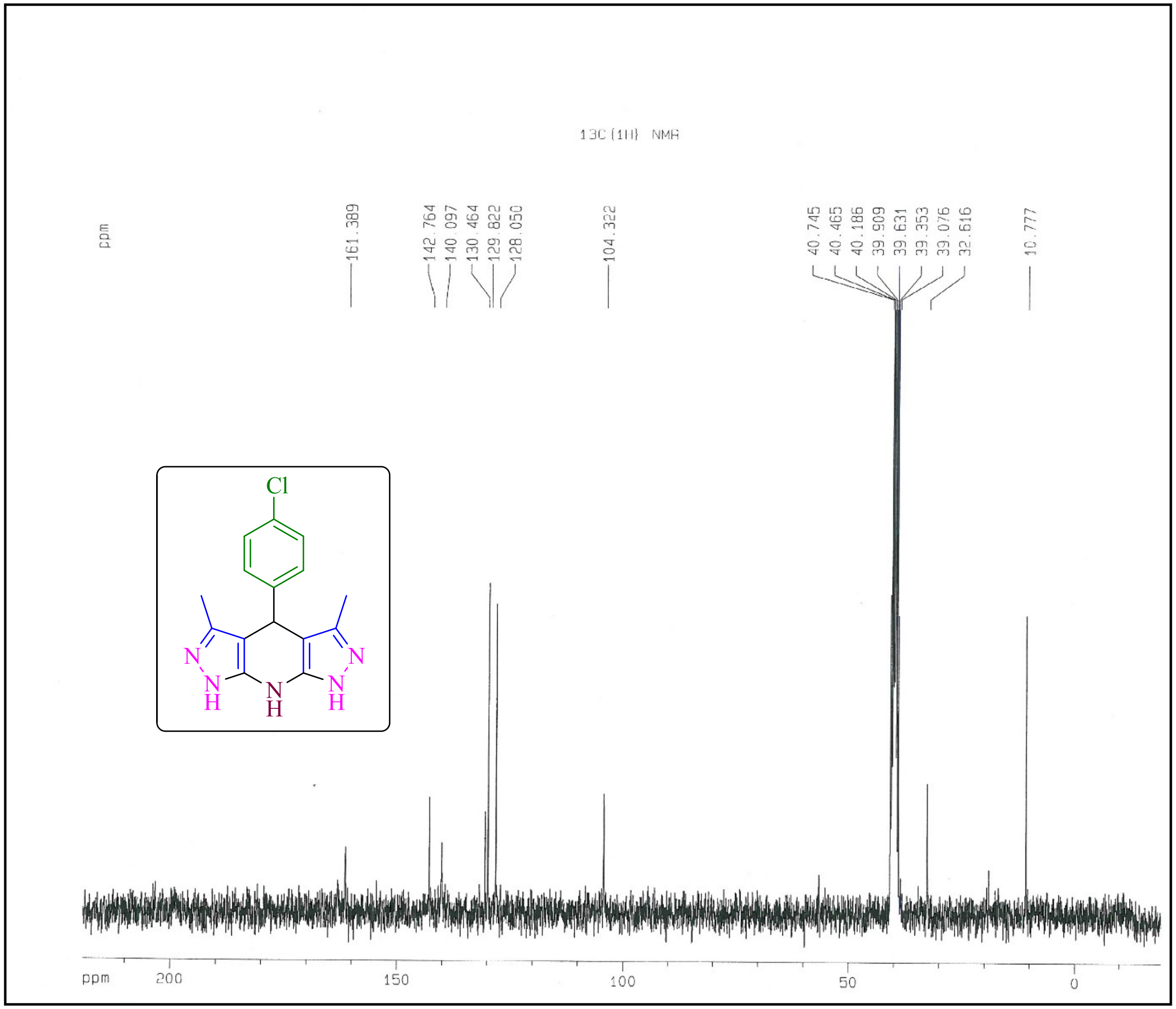

Figure S5. ^{13}C NMR spectrum of compound (**5d**)

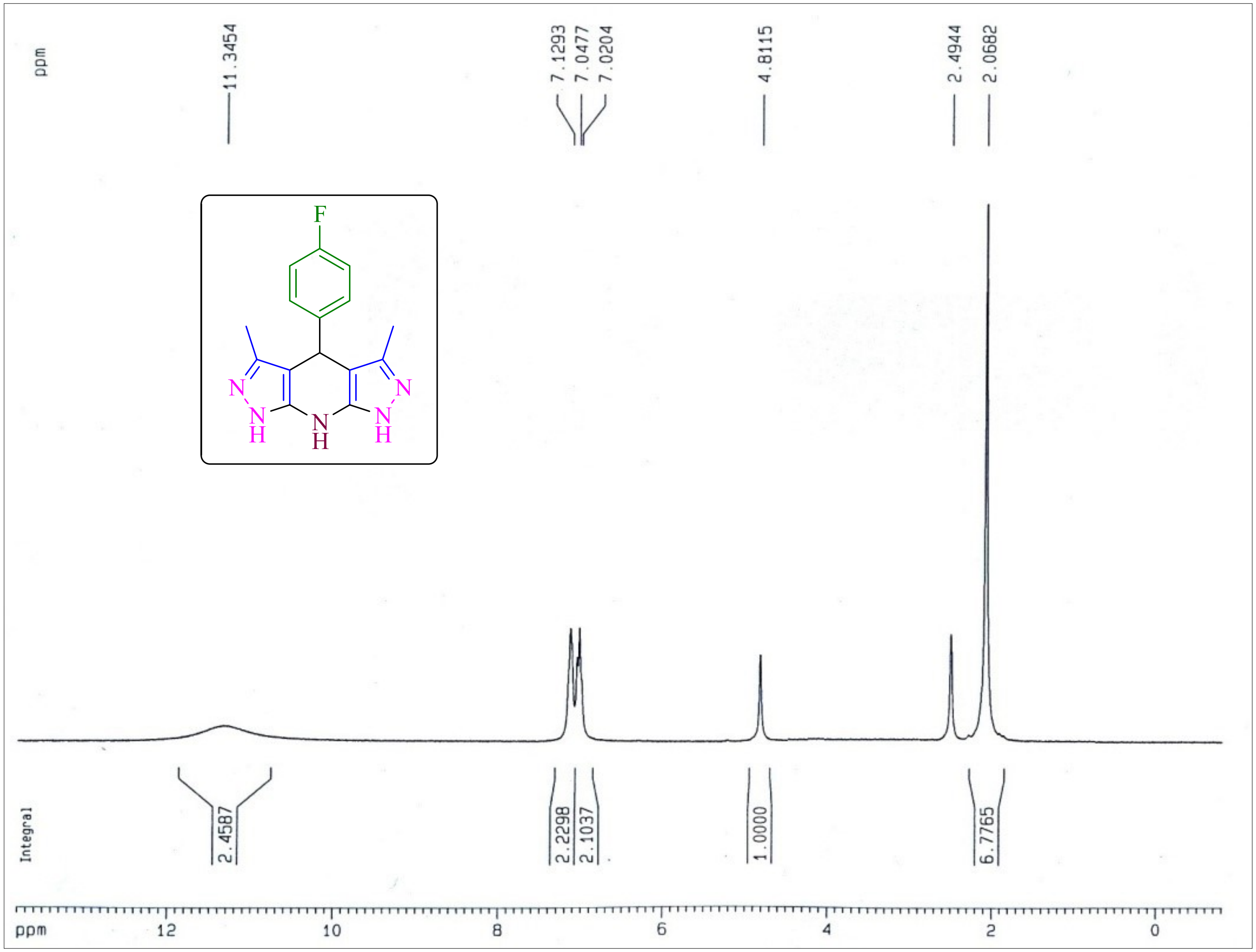

Figure S6. [13]H NMR spectrum of compound (**5j**)

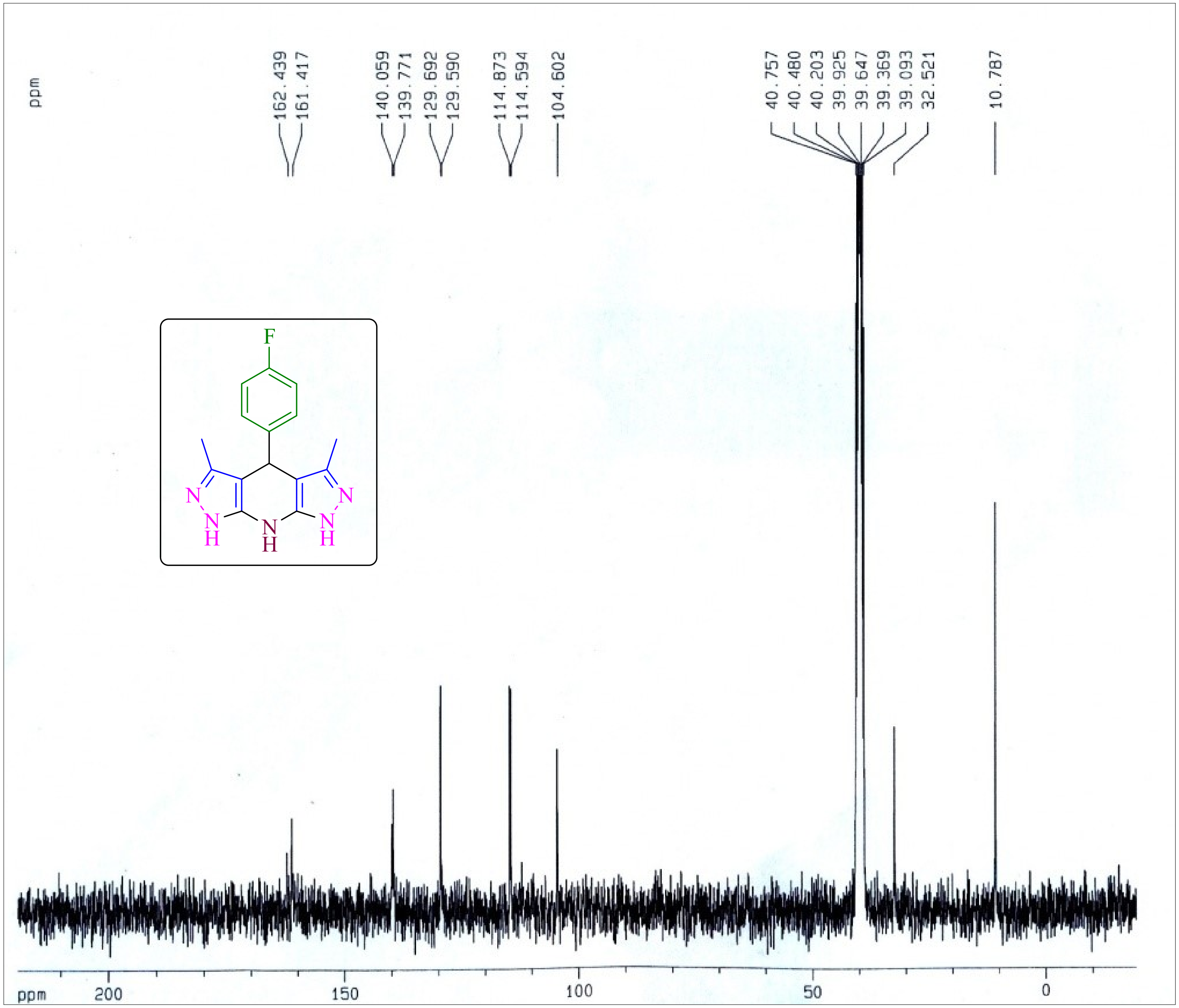

Figure S7. ^{13}C NMR spectrum of compound (**5j**)

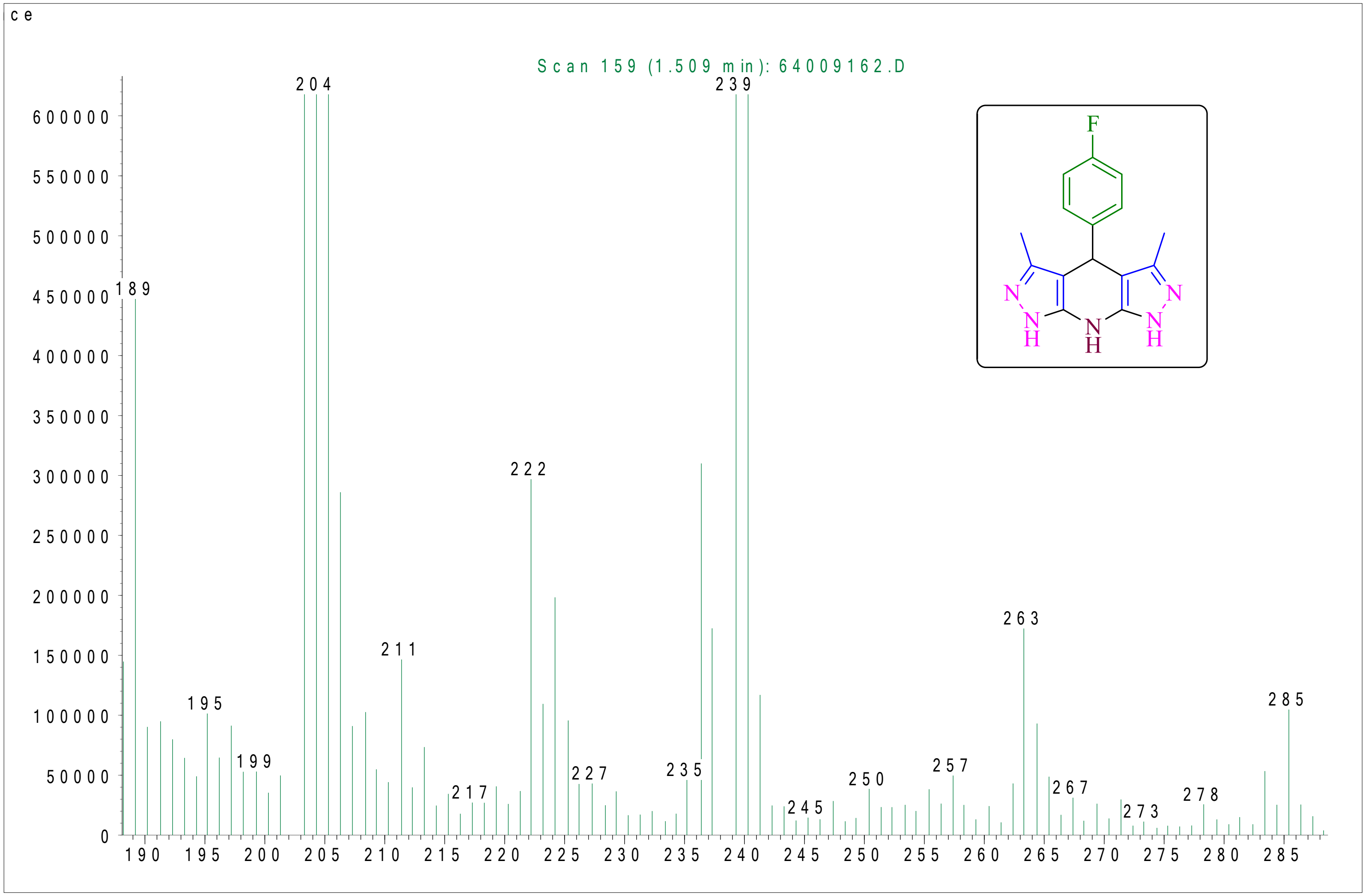

Figure S8. Mass spectrum of compound (**5j**)
